# Effect of physical parameter differences on the performance of a knowledge-based partial arc VMAT RapidPlan model for left breast cancer

**DOI:** 10.3389/fonc.2025.1589270

**Published:** 2025-05-13

**Authors:** Hai-liang Guo, Zeng-hong Lu, Jing-hua Zhong, Huai-wen Zhang

**Affiliations:** ^1^ Department of Oncology, the First Affiliated Hospital of Gannan Medical University, Jiangxi Clinical Research Center for Cancer, First Clinical Medical College, Gannan Medical University, Ganzhou, China; ^2^ Department of Radiation Oncology, Jiangxi Cancer Hospital & Institute, Jiangxi Clinical Research Center for Cancer, The Second Affiliated Hospital of Nanchang Medical College, Nanchang, China

**Keywords:** left breast cancer, DVH, RapidPlan, partial arc VMAT, dosimetry

## Abstract

**Objective:**

To optimize the protection of organs at risk (OARs) in left breast cancer radiotherapy, this study investigated how physical parameter adjustments affect the performance of a Rapidplan-based dose-volume histogram (DVH) prediction model.

**Methods:**

Twenty patients who underwent left breast-conserving surgery were enrolled. Partial arc volumetric modulated arc therapy (VMAT) plans were designed per patient, with X-direction field width set to half-beam and right breast (Breast-R) contoured as an avoidance structure to generate Rapidplan model. The model was used to predict and generate three plans: AP_partial arc (avoidance structure prioritized), RP_partial arc (no avoidance structure), and FP_partial arc (expanded field width). Dosimetric comparisons against the original plan evaluated the impact of parameter selection.

**Results:**

AP_partial arc reduced mean doses of Breast-R, Heart, Lung-L, and Lung-R by 7.7 cGy, 9.8 cGy, 16.7 cGy, and 1.1 cGy, respectively (p < 0.05). Conversely, RP_partial arc increased mean dose of Breast-R by 66.3 cGy (p < 0.05). FP_partial arc raised V5 of Lung-L, V5 of Heart, and mean dose of Lung-L by 4.01%, 2.25%, and 36 cGy (p < 0.05).

**Conclusion:**

The knowledge-based partial arc model for rapid planning of left breast cancer accurately predicts the DVH of OARs. However, before performing dose prediction, physical parameters such as radiation field width and planned avoidance structures should be considered to reduce the risk of low-dose exposure volume to OARs and secondary cancer.

## 1 Introduction

Breast cancer is a common malignant tumor in women (1). After breast-conserving surgery, most patients need radiotherapy to reduce the local recurrence rate. Three-dimensional conformal radiotherapy (3D-CRT), intensity-modulated radiotherapy (IMRT), and volume-rotating intensity-modulated radiotherapy (VMAT) have been used to treat breast cancer. Many studies have confirmed that VMAT irradiation technology has significant dosimetric advantages in the treatment of breast cancer (2, 3). However, full or half arc VMAT plans usually increase the low-dose exposure volume of the contralateral lung and breast, which may increase the risk of secondary cancer (4–6). Previous studies compared 50–60° partial arc VMAT with full-arc or half-arc VMAT. Their research results showed that partial arc technology reduced the radiation dose and volume of radiation to OARs on the contralateral side (7). Fogliata et al. conducted a risk assessment of VMAT and 3D-CRT radiotherapy technology for secondary cancer of the contralateral breast. The results showed that partial arc VMAT was as good as 3D-CRT in avoiding parts, and the acute and late NTCP levels of the affected organs were reduced (8). Even if partial arcs are used for VMAT planning and design, extensive planning design time is needed, and the consistency of the dose distribution quality is poor.

RapidPlan (Varian Medical Systems, USA) is a knowledge-based planning (KBP) solution that builds a predictive model by extracting historical planning information (9). These models can prospectively estimate the DVH of all OARs contained in the training model according to the anatomical characteristics of any new patient. The RapidPlan was reported to reduce the radiation dose to OARs and also improve the efficiency of the plan design (10–12). RapidPlan has been commercially promoted and has been extensively tested in many clinical cases (including VMAT of the breast cancer) (13, 14). The Varian RapidPlan model trained on VMAT and supine orientation can be used for other techniques and orientations (15, 16). The RapidPlan model configurations can be shared and implemented across multiple centers with simple adaptations to local protocols (17, 18). A VMAT KBP model driven by plans performed on a conventional linear accelerator (LINAC) with 6 MV flattening filter (FF) beams was reported to provide high-quality plans performed with 6 MV flattening filter-free (FFF) beams on the new Halcyon^©^ LINAC (19).

However, mismatched physical parameters between the optimization scheme and the RapidPlan model may result in deviations of the final dose distribution from the initial prediction. Yusuke Sakai et al. (20) developed a knowledge-based RapidPlan model using 32 TrueBeam SI-VMAT plans (1 full arc + 3 non-coplanar partial arcs). When validating the model on the Halcyon system, significant DVH deviations were observed in low-dose regions (<9 Gy), with differences in gradient index, conformity index, and normal brain volumes receiving ≥12 Gy, ≥18 Gy, and ≥27 Gy. Similarly, Cagni et al. (21) reported discrepancies between RapidPlan-predicted and RapidArc-achieved DVHs when applying a tomotherapy-trained model to arc-based plans, particularly for Spinal cord doses. Fogliata et al. (22) further highlighted that inconsistent avoidance sector settings in breast cancer models led to systematic overestimation of contralateral breast and contralateral lung doses.

Although a lot of evidence that physical parameters (avoidance structures, collimator field width) critically influence breast cancer dose distributions (23–25), existing RapidPlan models rarely integrate these factors during prediction. To address this challenge, this study developed a left breast cancer partial arc VMAT RapidPlan model and evaluated how to adjust to avoid the impact of structure and field width on OAR dose prediction. The results of this study aim to guide the selection of clinical parameters to reduce the exposure dose to breast-R, heart, lung-L, and lung-R.

## 2 Methods

This retrospective study included 20 consecutive patients with early-stage breast cancer (pathological stage T1N0M0) on the left side who underwent breast-conserving surgery between January 2023 and December 2024. Inclusion criteria: a pathologically confirmed diagnosis of T1N0M0 left breast cancer, ipsilateral breast CTV volume ≤1000cm³, age 18-70 years, and irradiation only to the whole breast and tumor bed. Exclusion criteria: previous chest radiotherapy or active systemic diseases (such as coronary artery disease, connective tissue disease), radiotherapy contraindications (such as pregnancy, pacemaker implantation), and the need for additional regional lymph node irradiation. The study protocol was approved by the institutional ethics committee (number: LLSC-2024067).

The patient was fixed in the breast bracket and vacuum negative pressure pad (model R610-DCF1, Klarity Company, China), the head was centered, and the rod was lifted and held with both hands. Computed tomography (CT) scanning was performed in the free-breathing mode. OARs, such as the left lung (Lung-L), Heart, right breast (Breast-R), right lung (Lung-R), Spinal cord, and trachea, were contoured. The clinical target volume (CTV) was the total volume of breast tissue measured on CT with the help of line markers placed around the palpable breast tissue. PTV was expanded 5 mm on the basis of the CTV but did not include the Heart. PTV and CTV were retracted 5 mm from the skin and restricted backward to the anterior edge of the intercostal space.

The partial arc VMAT plan (partial arc plan) for each patient was optimized on a Varian VitalBeam LINAC with a 6-MV FF photon energy beam, Millennium 120 leaf MLC, and jaw tracking mode. A dose rate of 600 MU/min was used to deliver 42.56 Gy to the PTV in 16 fractions. The VMAT plan was optimized using the photon optimization (PO) mode with Eclipse 15.5 3D planning system, and dose calculation was performed using the Acros XB algorithm with a calculation grid of 2.5 mm. The isocenter of the radiation field was placed on the midpoint of the line between the medial boundary and the lateral boundary of the PTV in the median transverse section CT image of each patient’s PTV, according to a report by Boman et al. (26). Field width in the X direction used a half-beam to reduce the effect of the beam divergence angle on the healthy lung and breast. Four partial arcs were used to design each plan. The first partial arcs were rotated 160°–165° to 95°–100counterclockwise and the arcs were reversed, and the second partial arcs were rotated 280°–285° to 350°–355°clockwise and the arcs were reversed, with a collimator at an angle of 0–10° to ensure that the bottom edge of the jaw was parallel to the sternum alignment to reduce radiation to the ipsilateral lung. Breast-R was defined as the avoidance structure to reduce exposure. A 10 mm virtual bolus was used in the optimization design to open the MLC leaf in the air outside the target volume to compensate for reductions in the dose coverage in the target area caused by the patient’s respiratory movement, breast edema, or breast deformation, according to the method proposed by Rossi et al. (27). The virtual bolus was removed during the final dose calculation.

The RapidPlan optimization component consisted of three main parts: a modeling and training engine, an automatic constraint prediction module, and a new VMAT/IMRT optimization objective based on the quality of the historical data used for training. During the extraction phase, several anatomical and dosimetric features were obtained from the patient’s anatomical structures and plan. Each OAR was divided into sub-volumes based on its position relative to the field and target. During the training phase, principal component analysis was performed on the OAR volume within the field to identify the geometric features most correlated with a dosimetric principal component score of 1. A regression model using these two components was applied to obtain the regression between anatomical/geometric features and dosimetric features. The OAR region that did not belong to the “in-field” was modeled using a simple model as the mean and standard deviation to estimate the dose. The final estimated DVH was based on the combination of different subvolume partitioned parts. In model evaluation, the goodness of fit (regression) was determined by the coefficient of determination R2 and the mean chi-squared x2. Potential outliers were also evaluated.

**Figure 1:**
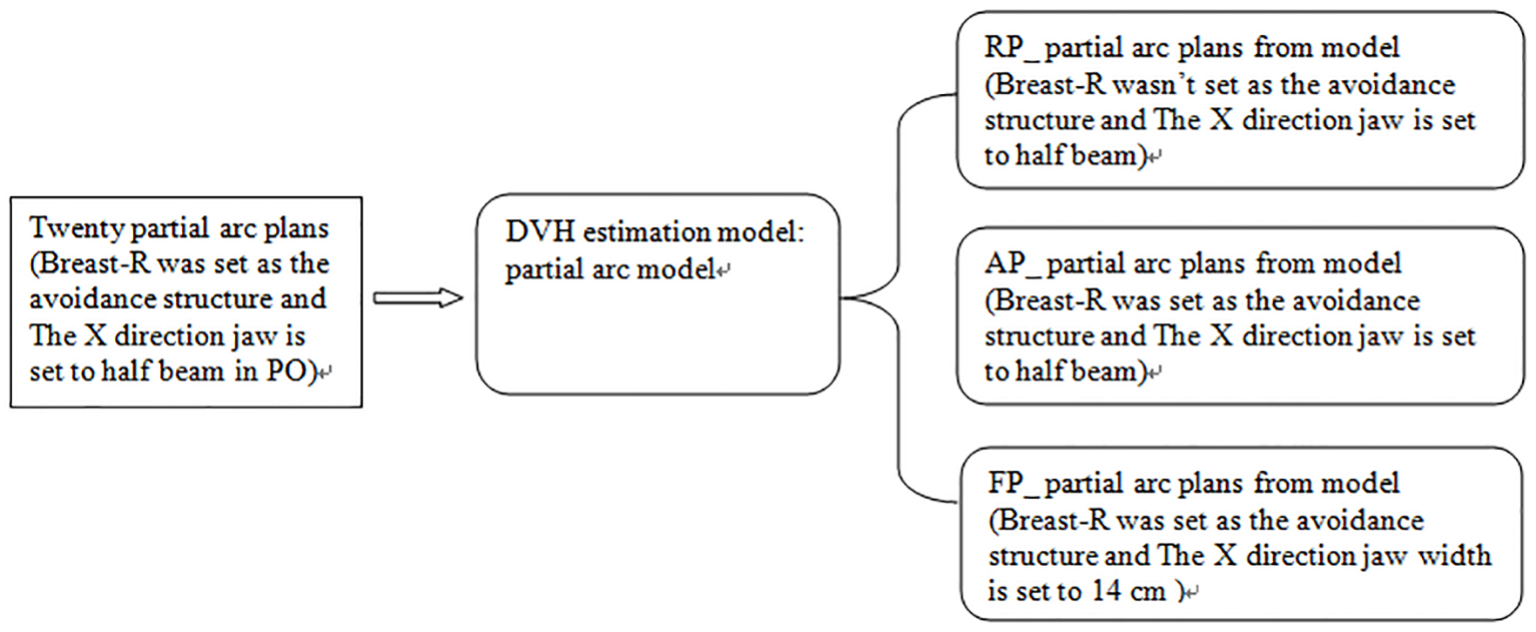
Diagram of the model and plans.

We used 20 partial arc plans created earlier to generate a fast-planning model, called the partial arc model, which considered radiation-endangered organs, including the Heart, Lung-L, Lung-R, and Breast-R. The selection of optimization objectives is shown in **Table 1**. Based on the guidelines provided by the manufacturer, this study observed various statistical charts to identify and classify possible geometric shapes, dose outliers, and points with strong influence in the fitted model after training. The thresholds for the modified Z-score (mZ), studentized residual (SR), and Cook’s distance (CD) were set at 3.5, 3.0, and 10.0, respectively. No samples exceeded these thresholds. Then, the model was used to generate dose distributions for 20 patients undergoing the optimization process, with the optimize goal determined by the model without any human modifications or interactions. This plan was called the RP_ partial arc plan. Then, the above operations were repeated, but Breast-R was set as the avoidance structure before optimization. This plan was called the AP_ partial arc plan. Finally, the width of the X-direction field was increased to 14 cm, and Breast-R was set as the avoidance structure. Then, the model was used to generate dose distributions on 20 patients during the optimization process, with the optimize goal determined by the model. This plan was named the FP_ partial arc plan, as shown in **Figure 1**.

**Table 1:** Optimization objectives in the RapidPlan model.

Structure	Parameter type	Objective	Priority
PTV	Upper Objective	D_max,0%_<102% of prescription	120
	Lower Objective	D_min,97%_>100% of prescription	120
	Lower Objective	D_min,100%_>99% of prescription	120
Breast-R	Line Objective	Generated	Generated
Heart	Line Objective	Generated	Generated
Lung-L	Line Objective	Generated	Generated
Lung-R	Line Objective	Generated	Generated
Spinal Cord	Line Objective	Generated	Generated

Finally, the output three plans of the RapidPlan model were compared with the original plan used to train the RapidPlan module of the left breast cancer. The comparison included various dose volume indicators, such as V95%, V107%, and D98% of PTV, and the mean dose of the Lung-L,Heart, Breast-R and Lung-R, V20 and V5 of Lung-L, V5 of Heart.

The dosimetric data were obtained through DVH. All of the DVH data were statistically analyzed using SPSS 16.0 software. The Shapiro-Wilk test was used to test the normality of the data, and the Levene test was used to verify the homogeneity of variance. The quantitative data are expressed as the mean ± standard deviation ( $\bar{x}\pm s$ ). The two related samples were compared via repeated measurement data analysis of variance. A p-value of < 0.05 indicated a statistically significant difference.

## 3 Results

### 3.1 partial arc VMAT RapidPlan model

**Figure 2:**
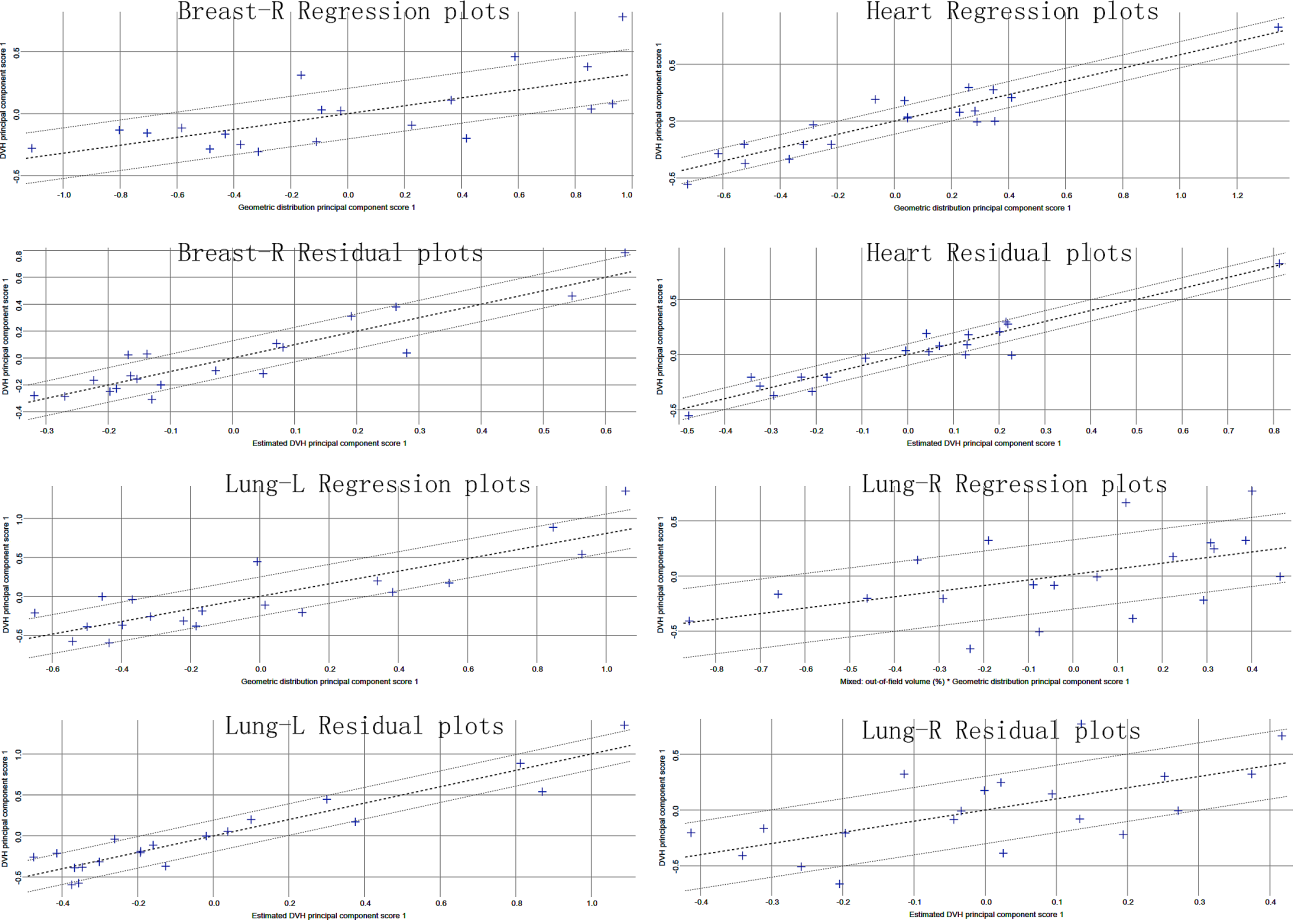
Regression and residual plots of the partial arc VMAT RapidPlan model.

**Table 2** reports the dosimetric characteristics of the partial arc plans used to generate the RapidPlan model. **Table 3** summarizes the model training results from the configuration information in terms of goodness-of-fit (coefficient of determination R^2^, chi-square x^2^, and outliers). All of the cases were accepted, and none was considered a true outlier. The regression plots and residual plots related to the four OARs are shown in **Figure 2**. Only Lung-R showed a large standard deviation and a large variance (dashed line). Other regression plots and residual plots had a clear slope, and the standard deviation and variance were small.

**Table 2:** Dose characteristics of the partial arc plans selected for model input.

Structure	Dosimetric goal	partial arc plans
PTV	D98%>95%[%]	97.70 ± 0.00[%]
	V107%<10%[%]	4.68 ± 2.30[%]
	V110%<1%[%]	0.14 ± 0.17[%]
Breast-R	Mean<400cGy[cGy]	71 ± 54[cGy]
Heart	Mean<500cGy[cGy]	213 ± 95[cGy]
	V_5Gy_<50%[%]	6.08 ± 3.98[%]
Lung-L	Mean<1000cGy[cGy]	576 ± 118[cGy]
	V_5Gy_<40%[%]	21.89 ± 4.16[%]
	V_20Gy_<20%[%]	10.36 ± 2.97[%]
Lung-R	V_5Gy_<20%[%]	0.00 ± 0.00[%]
	Mean<100cGy[cGy]	16 ± 8[cGy]

**Table 3:** Model training results.

Index	Breast-R	Heart	Lung-L	Lung-R
coefficients of determination R^2^	0.818	0.9	0.843	0.401
Chi-square x^2^	1.193	1.141	1.25	1.118
outliers	3	2	3	0

### 3.2 Comparison

**Figure 3:**
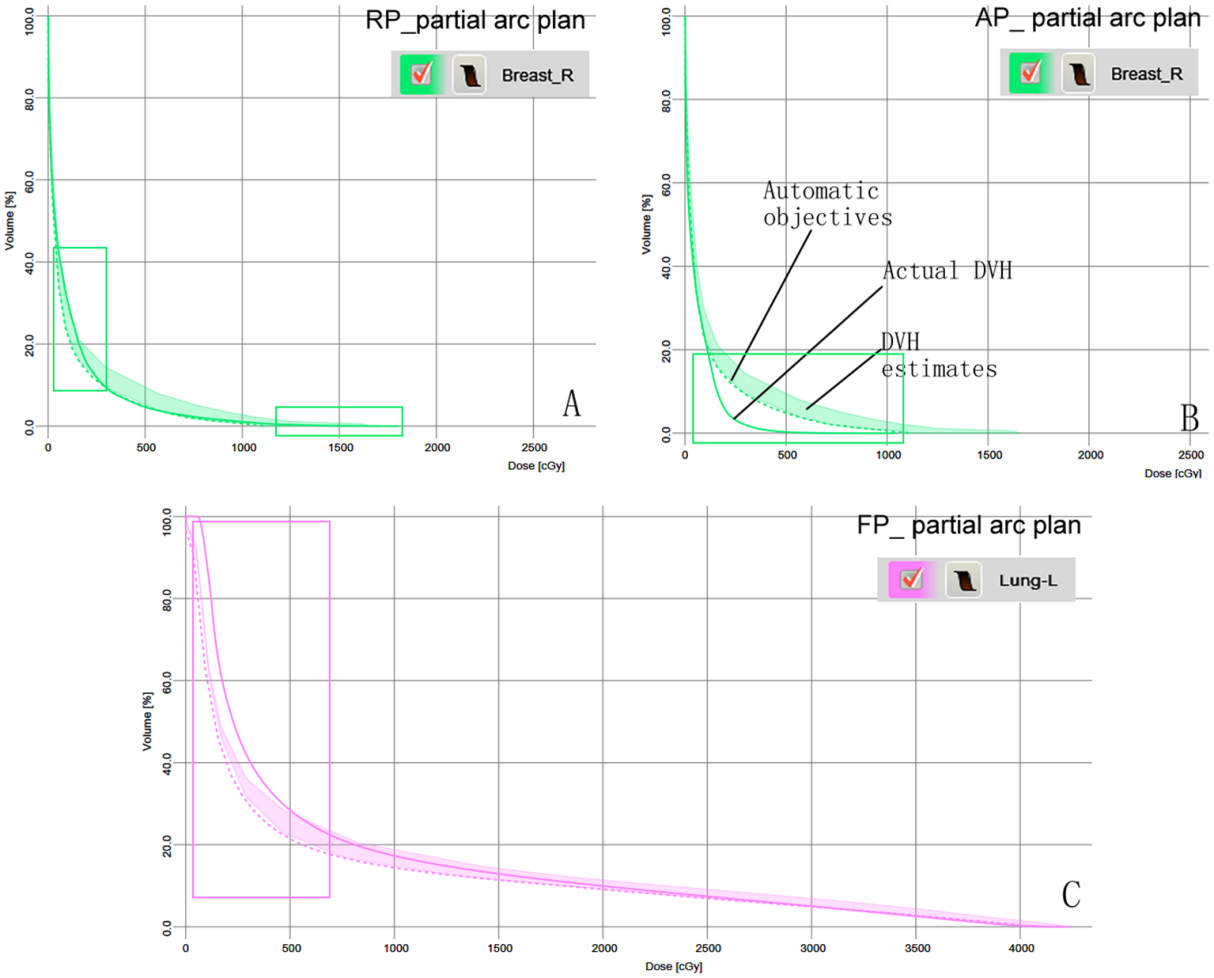
Model-based predictive objectives with the estimation range, automatic objectives (line objectives), and the actual DVH of patient 11 in the three plans. **(A)** Breast-R in the RP_ partial arc plan. **(B)** Breast-R in the AP_ partial arc plan. **(C)** Lung-L in the FP_ partial arc plan. The rectangular area represents the difference between the actual DVH and the predicted DVH.

**Figures 3A–C** shows the predicted DVH range and automatic objectives in the RapidPlan model based on KBP and the three sets of plans, as well as the predicted DVH difference between the actual and model-predicted DVH for patient 11.

**Figure 4:**
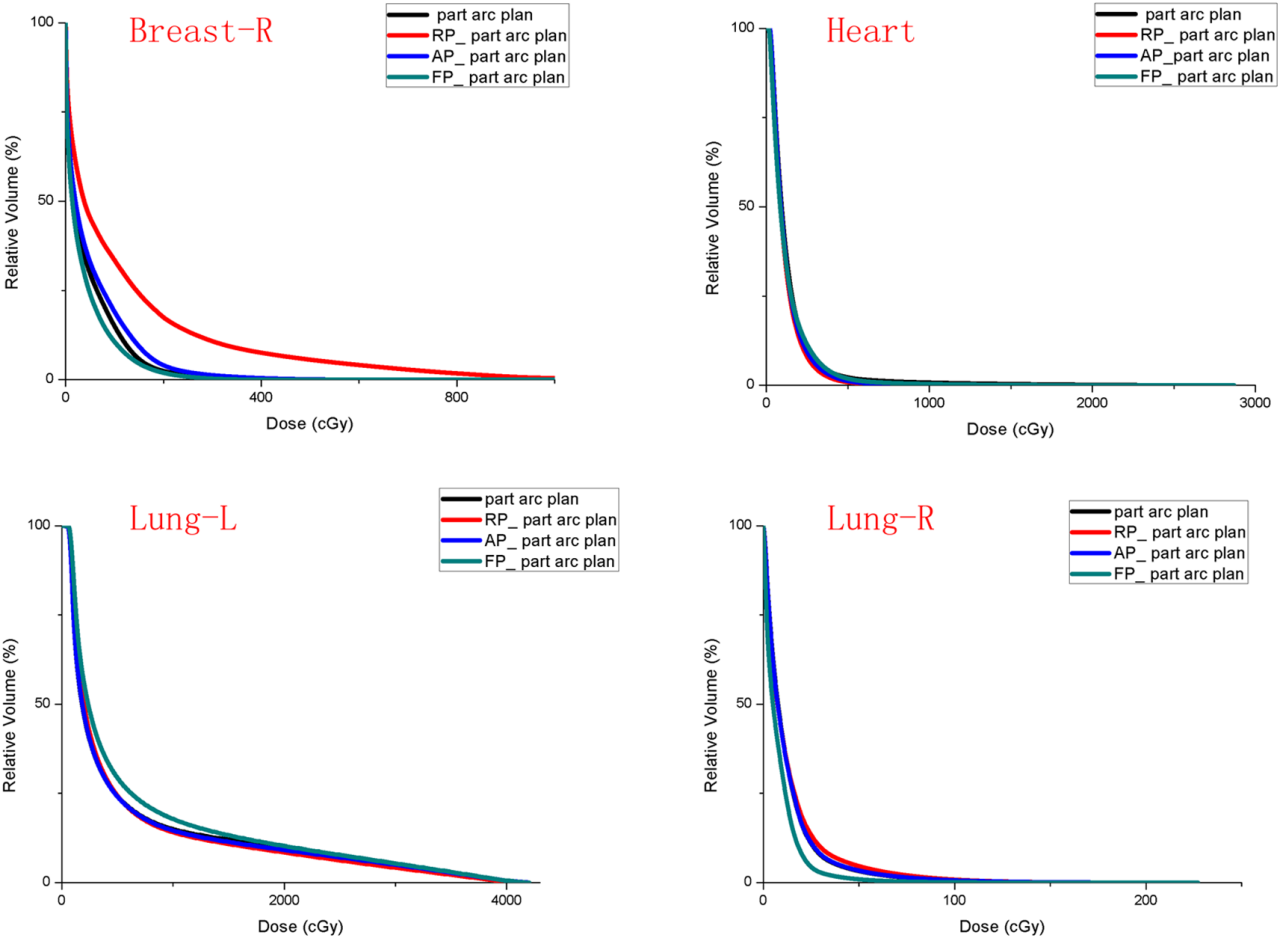
DVH of OARs comparison for patient 11 in the original plan and three plans generate by model.

**Figure 4** shows a DVH comparison of the original plan and the partial arc Model output three kinds of plans for Patient 11. The Breast-R DVH curve in the RP_ partial arc plan without Breast-R as an avoidance structure moved forward significantly. The Low-dose region of the Lung-L DVH curve in the FP_ partial arc plan moved forward, but Lung-R moved backward. The other cases in the cohort presented features similar to those of patient 11.

**Table 4** shows the comparison of doses to PTV and OARs between the original plan and the other three output plans. All three plans output by the partial arc model met the clinical prescription dose requirements and showed no difference from the original plan (p > 0.05). The AP_partial arc plan significantly reduced the average doses to breast-R, heart, lung-L, and lung-R, by 7.7 cGy, 9.8 cGy, 16.7 cGy, and 1.1 cGy, respectively (p < 0.05). Aligning the physical parameter settings with the RapidPlan model configuration improved the preservation of OARs compared to the original clinical plan. The study results showed that the RP_partial arc plan reduced the V5 values for lung-L and heart, but increased the average dose to breast-R to 66.3 cGy (p < 0.05). An increase in breast-R dose may increase the risk of secondary malignancies, such as contralateral breast cancer, by approximately 1.5% per Gy. The FP_partial arc plan resulted in an increase of 4.01%, 2.25%, and 36 cGy in V5 for lung-L, V5 for heart, and average dose to lung-L, respectively (p < 0.05). However, V5 of lung-L is one of the key indicators for predicting radiation pneumonitis, and an increase in low-dose (V5) volume may lead to an increased probability of radiation pneumonitis.

**Table 4:** Dose comparison of the PTV and OARs in the original plan and three plans generate by model.

Structure	Index	partial arc plan	RP_ partial arc plan	AP_ partial arc plan	FP_ partial arc plan
PTV	V95% (%)	99.3 ± 0.2	99.3 ± 0.2	99.2 ± 0.3	99.2 ± 0.2
	V107% (%)	4.8 ± 2.8	4.3 ± 2.6	4.3 ± 2.5	4.1 ± 2.6
	D98% (cGy)	4161.3 ± 13	4162 ± 13.8	4159.9 ± 15.8	4159.4 ± 15.6
Breast-R	mean dose (cGy)	71 ± 54	137.3 ± 113.0 *	63.3 ± 47.1 *	61.1 ± 44
Heart	mean dose (cGy)	213 ± 95	191.3 ± 92.6*	203.2 ± 96*	237.1 ± 101.4
	V5 (%)	6.08 ± 3.98	5.3 ± 3.8*	5.7 ± 3.9*	8.3 ± 5.5*
Lung-L	mean dose (cGy)	576 ± 118	543.3 ± 104.6*	559.3 ± 115.6*	612 ± 128.4*
	V5 (%)	21.89 ± 4.16	21.7 ± 3.8*	21.8 ± 4.2*	25.9 ± 5.6*
	V20 (%)	10.36 ± 2.97	9.4 ± 2.8*	10 ± 3*	10.3 ± 3.0*
Lung-R	mean dose (cGy)	16 ± 8	15.3 ± 8.5	14.9 ± 7. 0*	15.5 ± 7.7

*Statistically significant difference (p < 0.05) in pairwise comparisons compared to the partial arc plan.

## 4 Discussion

In the RapidPlan model established through the partial arc VMAT in this study, regression plots of four organs showed correlations between geometric and dosimetric features (**Figure 2**). Although Lung-R showed a large regression standard deviation and residual, the average dose to Lung-R was less than 20 cGy, and the data fluctuations were not clinically significant. All three sets of plans generated by the RapidPlan model met the clinical requirements (p > 0.05).

Most studies on the performance of the RapidPlan model utilize historical patient data to construct the RapidPlan model and employ it to re-optimize the MLC sequence while retaining other parameters such as field geometry and photon energy, subsequently comparing it with manual artificial plans (28). Unlike traditional methods, this study specifically focuses on two under-researched physical parameters—avoidance structure definition and collimator field width—to evaluate their impact on the performance of the RapidPlan model in left breast VMAT planning. The AP_partial arc plan replicates the physical parameters of the model training data, including Breast-R as an avoidance structure. When the structure is positioned before the target in the beam’s-eye-view or projected into the beam’s-eye-view in the Eclipse photon optimizer (as a different option), the closed MLC shields the avoidance structure, thereby reducing the dose received by the avoidance structure. Compared to manual plans, the AP_partial arc plan significantly reduces OAR doses (such as Dmean for the heart: 9.8 cGy, Dmean for Breast-R: 7.7 cGy, V20 for Lung-L: 0.36%, Dmean for Lung-L: 16.7 cGy) (p < 0.05). These findings indicate that RapidPlan’s ability in OAR protection is comparable to or even superior to manual planning, aligning with previous research results (14, 29) and supporting its clinical application in standardized left breast VMAT. Notably, the average doses for Lung-R and Breast-R in this study (Lung-R: 14.9 cGy; Breast-R: 63.3 cGy) were 15-20% lower than values reported in similar RapidPlan breast studies (30, 31). This may primarily be attributed to the synergistic effects of the partial arc VMAT geometry (limiting contralateral exposure) and strict avoidance structure implementation.

Radiation exposure to the contralateral breast is a recognized risk factor for secondary cancer during or after radiotherapy (32, 33). For women aged <40 years who received a contralateral breast dose >1.0 Gy, the long-term risk of secondary cancer was increase in a dose-dependent manner, which is inversely correlated with the age at exposure (34). In the RP_partial arc protocol, the average dose to Breast-R reached 1.37 Gy, exceeding the 1 Gy threshold associated with an increased risk of secondary cancer in younger patients. Compared to the original plan, the average dose to Breast-R increased by 66 cGy was potentially increasing the risk of secondary cancer in the contralateral breast by approximately 2.0%. This dose increase may be due to the fact that Breast-R was not considered as an avoidance structure during the optimization process. Although the partial arc rapid planning model generated automatic optimization targets, the lack of explicit Breast-R avoidance constraints resulted in unintended dose spillover. **Figure 3A** also shows that the actual dose volume histogram (DVH) cut-off dose for Breast-R is higher than the model prediction. This discrepancy reflects a mismatch between the avoidance structure configuration in the clinical plan and the parameters embedded in the rapid planning model, impairing its ability to perform contralateral breast avoidance. Antonella et al. (22) emphasized that model training requires strict alignment between the clinical plan and the avoidance structure defined in the RapidPlan configuration. Differences in these parameters will inevitably lead to systematic deviations between the predicted dose volume histogram (DVH) and the actual obtained DVH. Our research results also confirm this viewpoint, highlighting the necessity of coordinating the physical parameter settings (avoidance structure) with the model training framework to ensure reliable dose prediction and OAR protection.

The Eclipse treatment planning system employs jaw tracking technology in VMAT, where the jaw dynamically follows the MLC position to minimize inter-leaf leakage and reduce scatter dose to adjacent OARs (35). Different jaw widths significantly affect the dosimetry and complexity of VMAT plans. To balance modulation efficiency and delivery accuracy, it is generally recommended to set the starting position of the jaw as the target volume for automatic conformal, or to limit the jaw width in the X direction to ≤14 cm (36, 37). The original model training plan uses half-beam blocks in the X direction to minimize the impact of beam divergence on contralateral lung and breast tissue. In the FP_partial arc plan, the field width in the X direction is expanded to 14 cm while retaining the breast-R as an avoidance structure. Compared to the original plan, there was no significant change in the average dose of breast-R or lung-R. However, the V5 of lung-L increased by 4.01%, and the average dose of lung-L increased by 36 cGy (p < 0.05), which may be due to the following three factors: (1) the RapidPlan model may not accurately predict leakage outside the main beam, (2) increased MLC leaf travel distance leads to increased MLC leakage dose, and (3) prolonged MLC leaf travel time leads to prolonged beam on time. Yilmaz et al. (38) noted that the V5 of lung was an important predictor of radiation pneumonitis, and low-dose high-volume lung radiation causes greater damage to lung function than high-dose low-volume lung radiation. Recht A et al. studied radiation-induced lung injury caused by breast cancer radiotherapy and reported that the risk of radiation pneumonitis caused by relatively low-dose lung volume (V5) exposure after radiotherapy is significant (39). Rodrigues et al. also proposed that the Dmean of the ipsilateral lung is an important parameter for predicting radiation pneumonitis after radiotherapy (40). The FP_partial arc plan increases the V5 and Dmean of lung-L by 4% and 36 cGy, respectively, which may increase the probability of patients developing radiation pneumonitis. Similarly, an increase in the V5 and average dose of the heart (2.1%, 24 cGy) may lead to long-term cardiac toxicity. Darby et al. demonstrated that a 1 Gy increase in major coronary artery events linearly increases by 7.4% (41), emphasizing the need to minimize cardiac exposure even at low doses. Overall, these results highlight that deviations in field width settings and model training parameters can compromise its ability to limit low-dose exposure to critical organs, especially the heart and ipsilateral lung.

Breast radiotherapy dose distribution is influenced by various factors, including the angle/number of rotational arcs, collimator angle, breast CTV shape/size, and non-coplanar field configuration. This study focuses solely on the impact of avoidance structures and field width on the performance of the RapidPlan model. This study is based on data from 20 patients at a single center, and the field width was fixed during the training of the KBP model, which limits our ability to study the model’s generalization performance, especially for patients with larger breast volumes (>1000 cm³) or complex geometric shapes. In future research, we will expand the KBP training dataset by collecting data from breast cancer patients at multiple radiotherapy centers, while fully considering changes in arc angles (e.g., 180°–300°), collimator rotation (15°–45°), breast CTV volume (500–1500 cm³), and the definition of avoidance structures to enhance the model’s adaptability to heterogeneous physical parameters. Referring to Baroudi et al., a hybrid AI architecture can be explored: the nnU-Net model can autonomously optimize the gantry angle and field shape based on the spatial relationship between breast CTV/OAR, while RapidPlan generates dose targets (42). This integration will standardize plan quality by reducing operator-dependent variability.

## 5 Conclusions

The knowledge-based partial arc model for rapid planning of left breast cancer accurately predicts the DVH of OARs. However, before performing dose prediction, physical parameters such as radiation field width and planned avoidance structures should be considered to reduce the risk of low-dose exposure to OARs and secondary cancer.

## Data Availability

The original contributions presented in the study are included in the article/supplementary material. Further inquiries can be directed to the corresponding author.
